# Supplementary material for: Intragenic Deletions in FLNB Are Part of the Mutational Spectrum Causing Spondylocarpotarsal Synostosis Syndrome
Source: Genes (Basel). 2021 Apr 5;12(4):528. doi: 10.3390/genes12040528 (PMC8065484; doi:10.3390/genes12040528)
Supplement: Supplementary file 1 [file genes-12-00528-s001.pdf]

Supplementary Information

Table S1. Primer sequences used for the allele specific PCR reactions.

Target	Set	Primer Sequences
Family A<br>6432 bp deletion<br>(exons 9-12)	Set 1 (WT, 933 bp)	F: TTTCCACCATATAACCAGGGAGAC<br>R: GATCCCCGGAGTTGCAAGAA
	Set 2 (Mut, 262 bp)	F: CAGCTTCTGCTTCCCTGGTTC<br>R: GTGCTAAGTGCTACTACCAG
Family B<br>6166 bp deletion<br>(exons 21-24)	Set 1 (WT, 444 bp)	F: CACTTGCGTTAGGAGAGAGTTGG<br>R: CCCCCATAGGTTGCCTTAGC
	Set 2 (Mut, 178 bp)	F: GAGCAAATTGCAACAGCCCA<br>R: CCCCCATAGGTTGCCTTAGC

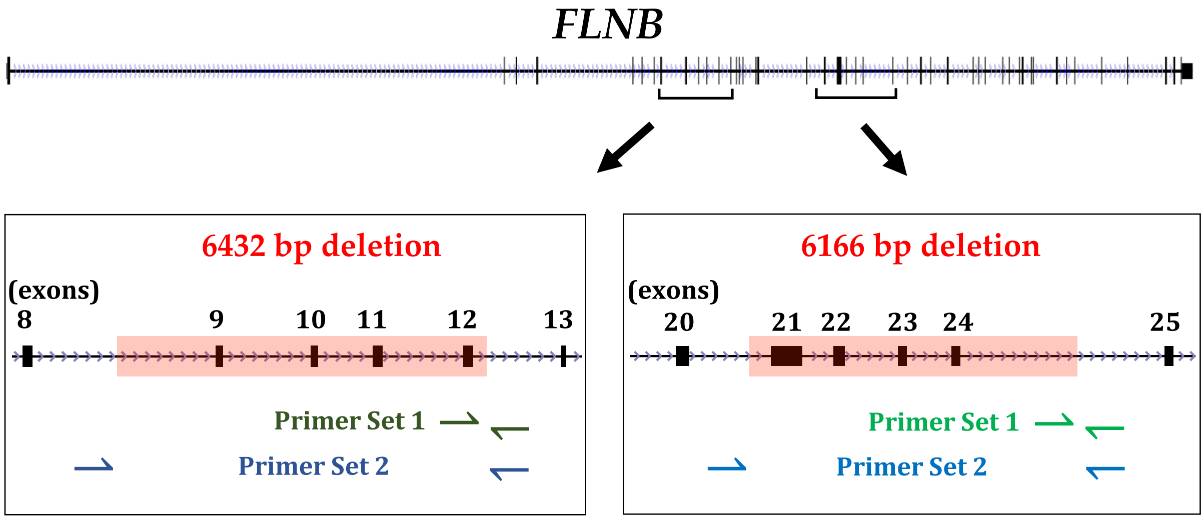

Figure S1. Design of the allele specific PCR reactions. At the top is the structure of the *FLNB* gene with vertical bars indicating exons. Lower left shows a zoom in of the region containing the 6432 bp deletion encompassing exons 9-12 which was found in family A. Lower right shows the 6166 bp deletion encompassing exons 21-24 which was found in family B. In both pictures, the positions of the primers are indicated using harpoon arrows. Set 1 was designed with the forward primer within the deletion so that amplification only occurs from the wild-type allele. Set 2 was designed with both primers outside the deletion so that amplification only occurs from the deletion-containing allele since any products from the wild-type allele would be too large for the cycling conditions used.
